# Diagnostic accuracy of quantitative PCR (Xpert MTB/RIF) for tuberculous pericarditis compared to adenosine deaminase and unstimulated interferon-γ in a high burden setting: a prospective study

**DOI:** 10.1186/1741-7015-12-101

**Published:** 2014-06-18

**Authors:** Shaheen Pandie, Jonathan G Peter, Zita S Kerbelker, Richard Meldau, Grant Theron, Ureshnie Govender, Mpiko Ntsekhe, Keertan Dheda, Bongani M Mayosi

**Affiliations:** 1 The Cardiac Clinic, Department of Medicine, Groote Schuur Hospital and University of Cape Town, Groote Schuur Drive, Observatory, Cape Town 7925, South Africa; 2 Lung Infection and Immunity Unit, Division of Pulmonology and UCT Lung Institute, Department of Medicine, Groote Schuur Hospital and University of Cape Town, Cape Town, South Africa; 3 TB Vaccine Group, Jenner Institute, University of Oxford, Oxford, UK; 4 Institute of Infectious Diseases and Molecular Medicine, University of Cape Town, Cape Town, South Africa

**Keywords:** Tuberculous pericarditis, Adenosine deaminase, Interferon γ, Xpert MTB/RIF test, Diagnosis

## Abstract

**Background:**

Tuberculous pericarditis (TBP) is associated with high morbidity and mortality, and is an important treatable cause of heart failure in developing countries. Tuberculous aetiology of pericarditis is difficult to diagnose promptly. The utility of the new quantitative PCR test (Xpert MTB/RIF) for the diagnosis of TBP is unknown. This study sought to evaluate the diagnostic accuracy of the Xpert MTB/RIF test compared to pericardial adenosine deaminase (ADA) and unstimulated interferon-gamma (uIFNγ) in suspected TBP.

**Methods:**

From October 2009 through September 2012, 151 consecutive patients with suspected TBP were enrolled at a single centre in Cape Town, South Africa. *Mycobacterium tuberculosis* culture and/or pericardial histology served as the reference standard for definite TBP. Receiver-operating-characteristic curve analysis was used for selection of ADA and uIFNγ cut-points.

**Results:**

Of the participants, 49% (74/151) were classified as definite TBP, 33% (50/151) as probable TBP and 18% (27/151) as non TBP. A total of 105 (74%) participants were human immunodeficiency virus (HIV) positive. Xpert-MTB/RIF had a sensitivity and specificity (95% confidence interval (CI)) of 63.8% (52.4% to 75.1%) and 100% (85.6% to 100%), respectively. Concentration of pericardial fluid by centrifugation and using standard sample processing did not improve Xpert MTB/RIF accuracy. ADA (≥35 IU/L) and uIFNγ (≥44 pg/ml) both had a sensitivity of 95.7% (88.1% to 98.5%) and a negative likelihood ratio of 0.05 (0.02 to 0.10). However, the specificity and positive likelihood ratio of uIFNγ was higher than ADA (96.3% (81.7% to 99.3%) and 25.8 (3.6 to 183.4) versus 84% (65.4% to 93.6%) and 6.0 (3.7 to 9.8); *P* = 0.03) at an estimated background prevalence of TB of 30%. The sensitivity and negative predictive value of both uIFNγ and ADA were higher than Xpert-MT/RIF (*P* < 0.001).

**Conclusions:**

uIFNγ offers superior accuracy for the diagnosis of microbiologically confirmed TBP compared to the ADA assay and the Xpert MTB/RIF test.

## Background

Tuberculosis (TB) is a global health priority [1]. In developing countries with dual human immunodeficiency virus (HIV) and TB epidemics there continues to be high TB-related mortality [2]. In immunosuppressed patients, this high mortality can be largely attributed to the increased burden of disseminated and severe forms of extra-pulmonary TB, such as tuberculous pericarditis (TBP) [3]. TBP carries a high case fatality rate (17% to 40% over six months) [3] and accounts for approximately 7% of hospital admissions for acute heart failure in Africa [4]. Despite the burden of disease and associated high mortality, the diagnosis of TBP remains problematic because of the lack of a simple, rapid, accessible and accurate diagnostic test [5]. TBP fluid is known to be paucibacillary with estimated culture and microscope smear-based diagnostic accuracy of only approximately 50% and 5%, respectively [6]. A definitive diagnosis of TBP is, therefore, challenging and often delayed [7]. Recent studies have indicated that the rapid initiation of anti-TB treatment may reduce mortality, making the investigation of new, rapid diagnostic tests for TBP essential [8].

The Xpert MTB/RIF assay is a new quantitative polymerase chain reaction (PCR) test that has been introduced for the rapid diagnosis of *Mycobacterium tuberculosis* (*M. tb*) and rifampicin resistance, providing a result in less than two hours [9]. Xpert MTB/RIF is endorsed by the World Health Organization (WHO) for the diagnosis of pulmonary TB using sputum samples [10]. Validation studies using culture positive sputum samples from pulmonary TB patients show a pooled sensitivity of 98% and 68% in smear-positive and -negative cases, respectively, and an overall pooled specificity of 98% [11]. Except for a few isolated cases, there are no prospective studies of the diagnostic utility of Xpert MTB/RIF test in TBP [12,13].

By contrast, proof-of-principle studies have demonstrated the potential utility of the novel biomarker, unstimulated interferon gamma (uIFNγ) as a diagnostic tool in pericardial and pleural fluid [14,15]. One study found that, when using a diagnostic cut-point of 0.2 IU/ml, pericardial fluid uIFNγ offered a 98% sensitivity and 100% specificity for the diagnosis of TBP. Despite these promising early results, the measurement of uIFNγ has not translated into routine clinical practice partly because of the lack of validation of the original observations [5].

Adenosine deaminase (ADA) level is the current locally available surrogate measure that suggests *M. tb* infection. The South African National Health Laboratory Service (NHLS) reference ranges for normal ADA levels are: 0 to 15 U/L for serum, 0 to 30 U/L for pleural fluid and 0 to 9 U/L for cerebrospinal fluid. Locally available, yet unvalidated data regarding ADA measurements in pericardial fluid suggests an ADA cut-off value of 40 U/L resulted in a test sensitivity, specificity, positive predictive value, negative predictive value and diagnostic efficiency of 84%, 80%, 91%, 66%, and 83%, respectively [16].

The aim of this study was to assess the diagnostic utility of the new Xpert MTB/RIF test compared to ADA and uIFNγ assays in the diagnosis of TBP in a population with a high burden of TB.

## Methods

### Study population

Between October 2009 and September 2012, consecutive patients with suspected TBP referred to Groote Schuur Hospital in Cape Town for enrolment in the Investigation of Management of Pericarditis in Africa (IMPI Africa) registr*y*[17] were screened for inclusion in this diagnostic study. Inclusion criteria were the presence of a large pericardial effusion amenable to safe pericardiocentesis (greater than 10 mm echo-free space around the heart in diastole), age 18 years or older and the provision of informed consent. Exclusion criteria were pregnancy, anti-TB treatment initiation >1 week prior to pericardiocentesis and refusal or inability to sign consent. Informed consent was obtained from each patient prior to enrolment in the registry and the study protocol conforms to the ethical guidelines of the 2008 Declaration of Helsinki as reflected in *a priori* approval by the human research ethics committee of the University of Cape Town (HREC REF402/2005) (additional data provided in Additional file 1).

### Diagnostic sample collection and handling

A minimum of 60 ml of pericardial fluid (PF) was collected for diagnostic testing by means of percutaneous pericardiocentesis. PF was sent to the NHLS for measurement of ADA and lactate dehydrogenase (LDH) levels, differential cell counts and cytology, as well as routine TB diagnosis consisting of concentrated fluorescence smear microscopy and mycobacteria growth indicator tube (MGIT) liquid culture (MGIT 960, BD Diagnostics, Hunt Valley, MD, USA). Drug susceptibility testing was performed on positive culture isolates using the Genotype MDRTBplus assay (Hain Lifescience, Nehren, Germany). In addition, PF samples were stored at -20°C, for later measurement of uIFNγ levels and performance of the Xpert MTB/RIF assay. Investigators performing Xpert MTB/RIF and uIFNγ were blinded to clinical and routine TB diagnostic findings and categorisation (additional data provided in Additional file 1).

### Xpert MTB/RIF assay

The Xpert MTB/RIF assay was performed on PF samples using the manufacturer’s specifications for sputum samples as previously described (Cepheid, Sunnyvale, CA, USA) [9]. Where possible, Xpert MTB/RIF was performed using both 1 ml of unconcentrated and unprocessed PF as well as 3 to 20 ml of centrifuged (3,000 g × 15 minutes) PF reconstituted to 1 ml with phosphate buffered saline (PBS). The fourth generation Xpert MTB/RIF cartridge was used. The cycle threshold value (C_T_-values) indicates the cycle number at which the molecular probe becomes detectable and is proportional to the amount of TB-specific starting template. The average C_T_-value for the five TB-specific molecular probes and for the spore-related positive control (lyophilized *Bacillus atrophaeus* subsp. *globigii* spores) (SPC) are used as surrogate markers of bacillary load and PCR inhibition, respectively. All Xpert MTB/RIF results were available within two hours from the time of sample processing. The limit of detection was determined in duplicate by spiking 0, 50, 75, 100 and 150 H37Rv colony forming units (CFU) to 1 ml aliquots of PF before dilution with sample buffer and subsequent Xpert MTB/RIF analysis. This experiment was repeated twice, thus providing four replicates for each CFU concentration. Inhibition was evaluated by comparing the PCR cycle-threshold (C_T_) values of the SPC from unconcentrated and concentrated samples.

### ADA assay

An adenosine deaminase assay (Diazyme, Poway, CA, USA, [18]) was performed on 1 to 8 ml PF samples, collected in serum tubes, according to the manufacturer’s specifications by the National Health Laboratory Services, Groote Schuur, Cape Town (NHLS GSH). Samples were either processed immediately or stored (at 2 to 4°C) for processing within 24 hours.

The Diazyme ADA assay is based on the enzymatic deamination of adenosine to inosine, which is converted to hypoxanthine by purine nucleoside phosphorylase. The reagent is used at 37°C ± 0.5°C, using an instrument that is capable of reading absorbance accurately at 540 nm to 550 nm. ADA activity was measured as units per litre (U/L), where one unit of ADA is defined as the amount of ADA that generates one micromole (μmol) of inosine from adenosine per minute at 37°C.

### uIFNγ assay

uIFNγ levels were measured in duplicate using supernatant attained from 3 to 20 ml of thawed and centrifuged (3,000 g for 15 minutes) PF using the InterGam Ultrasensitive Rapid Immuno-suspension Assay (IRISA; Antrum Biotech, Cape Town, South Africa; http://www.antrumbiotech.com; limit of detection = 5 to 10 pg/ml) following the manufacturer’s instructions and without antigen stimulation.

### Diagnostic classification for analysis

All participants who were included had a large pericardial effusion on echocardiography. Participants were categorised into the following diagnostic groups based on a combination of pericardial and non-pericardial sample culture results, histopathology of pericardial biopsy samples, basic PF characteristics, and the commencement of TB treatment as follows: (i) Definite-TB: at least one *M. tb* sample positive by liquid culture (either pericardial or non-pericardial) and/or granulomatous inflammation on pericardial tissue histology (that is, composite reference standard); (ii) Probable-TB: not meeting the criteria for definite-TB, but based on clinical suspicion (symptoms, imaging, and preliminary fluid analysis) commenced empirically on TB treatment in the absence of an alternative diagnosis; (iii) Non-TB: no microbiological evidence of *M. tb* and an alternative diagnosis is available.

### Modelling clinical predictors using multiple imputation

A univariable analysis was used to determined basic clinical predictors of definite TBP. Thereafter, a set of multivariable clinical predictors was generated using logistic regression modelling. Multiple imputation by chained equations was used to impute missing data prior to model building [19]. Rounded ß-coefficients from the reduced model of significant variables were used to generate scores to quantitate relevant clinical predictors. Receiver operating characteristic (ROC) curve analysis was performed and three cut-points were selected for rule-in, Youden’s index (the optimal mathematical balance between sensitivity and specificity) [20] and rule-out value. Diagnostic accuracy, including 95% CIs, for each cut-point, was assessed. Performance was also compared against a previously formulated clinical prediction rule (Tygerberg TB Pericarditis Diagnostic Index Score (TDIS) of ≥6) [6].

### Statistical analysis

Sensitivity, specificity, positive (LR+) and negative (LR-) likelihood ratios, and positive predictive values (PPV) and negative predictive values (NPV) for all diagnostic tests are presented with 95% CIs. Demographic, clinical and microbiological characteristics of different groups were compared using *χ*^2^ and Wilcoxon rank-sum tests as appropriate. Diagnostic sensitivity and specificity of individual and/or combinations of tests were compared using the *χ*^2^ and Fisher’s exact tests as appropriate. The Spearman correlation coefficient (R_s_) was used to evaluate the association between Xpert MTB/RIF-generated PCR cycle-threshold (C_T_) values and liquid culture time-to-positivity. All statistical tests were two sided at α = 0.05. STATA IC, version 10 (Stata Corp, College Station, TX, USA) was used for all statistical analyses. The STARD criteria were used for analysis and reporting of this study [21].

## Results

### Clinical characteristics

**Figure 1:**
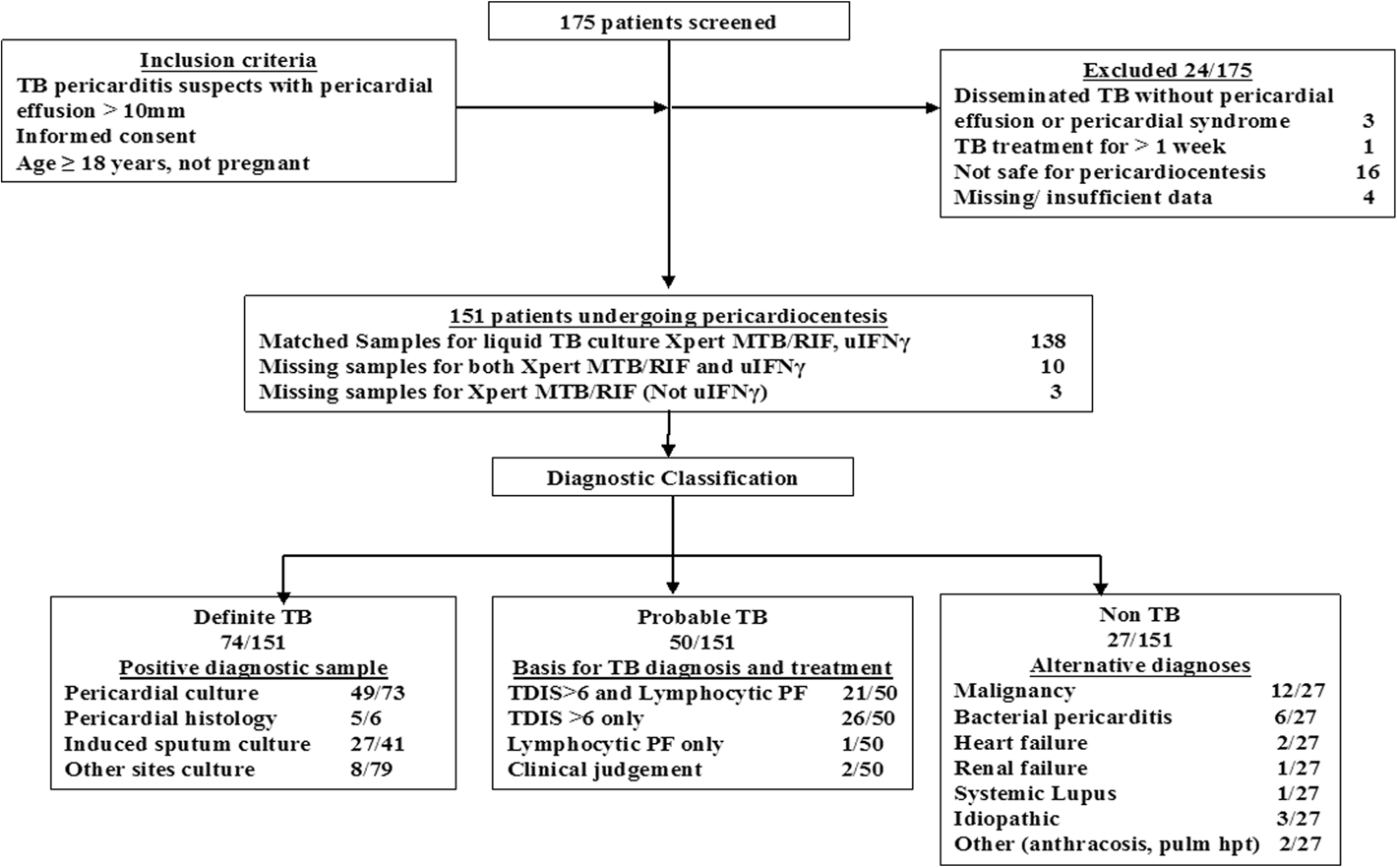
**Screening, recruitment and diagnostic classification of patients with suspected tuberculous pericarditis.** TB = tuberculosis; uIFNγ = unstimulated interferon gamma; TDIS: Tygerberg diagnostic index score; PF: pericardial fluid. *Insufficient clinical or diagnostic data acquired at baseline assessment.

Figure 1 shows the study flow chart. Of the 175 patients screened, 24 patients were excluded due to having pericardial effusions that were not amenable to safe pericardiocentesis (n = 16), missing information (n = 4), absence of a pericardial effusion (n = 3) and prolonged TB therapy (n = 1). Of the remaining 151 patients, 49.0% (74/151), 33.1% (50/151) and 17.9% (27/151) were classified as definite-, probable- and non-TB, respectively. Only 1/74 definite-TB patients was PF smear-positive.

Tables 1A and B show the clinical characteristics of patients with suspected TBP stratified by final diagnostic group. Of these patients, 74% (105/151) were HIV-infected with a median (interquartile range (IQR)) CD4 count of 139 (81 to 249); 9/151 participants refused HIV testing or had an unknown HIV status. Only 18% (18/98) of HIV-infected patients were on anti-retroviral therapy at enrolment. Non-TB participants were significantly older, less likely to be HIV-infected, and more likely to have severe shortness of breath despite significantly smaller pericardial effusions than those with definite and probable TB. In contrast, definite-TB and probable-TB patients had similar clinical characteristics.

**Table 1:** Baseline demographic and clinical (A), echocardiographic and biochemical (B) characteristics of patients referred with suspected TB pericarditis

**A.**				
**Baseline feature**	**All study patients**	**Definite-TB**	**Probable-TB**	**Non-TB**
	**Number = 151**	**Number = 74**	**Number = 50**	**Number = 27**
**Demographics**				
Age (median, IQR)	34 (29 to 42)	33 (27 to 38)^*^	33 (28 to 37)^*^	52 (34 to 60) ^*^P <0.001
Male (number, %)	93 (62)	48 (65)	32 (64)	13 (48)
HIV positive (number, %)^a^	105 (74)	59 (80)^*^	41 (82)^*^	5 (28) ^*^P < 0.001
CD4 count (median, IQR)^a^	139 (81 to 249)	131 (70 to 206)^*^	153 (81 to 271)^*^	301 (229 to 424) ^*^P = 0.04
ARV therapy (number/Number, %)	18/98 (18)	10/54 (19)	7/39 (18)	1/5(20)
**Presenting clinical features**				
NYHA Class I – II (number/Number, %)	77/134 (58)	39/66 (59)^*^	35/47 (75)^*^	3/21 (14) ^*^P < 0.001
NYHA Class III – IV (number/Number, %)	57/134 (41)	27/66 (41)^*^	12/47 (25)^*^	18/21 (86) ^*^P < 0.001
Systolic blood pressure (mean, SD) (number = 145)	113 (17)	113 (17)	112 (16)	115 (22)
Diastolic blood pressure (mean, SD) (number = 145)	72 (14)	72 (15)	72 (13)	73 (14)
Heart rate (mean, SD) (number = 146)	111 (20)	114 (22)	108 (16)	111 (21)
**Serum biochemical data**				
Haemoglobin g/dl (mean, SD)	9.6 (2.1)	9.4 (2.1)^*^	9.3 (1.7)^*^	10.9 (2.3) ^*^P = 0.004
Creatinine, μmol/L (median, IQR)	73 (59 to 90)	72 (58 to 86)	77 (61 to 92)	72 (61 to 81)
Total WCC × 10^9^/L (median, IQR)	6.5 (4.8 to 9.3)	6.5 (4.4 to 8.2)^*^	5.8 (4.8 to 7.2)^*^	10.5 (7.1 to 15.2) ^*^P <0.001
**B.**				
**Echocardiographic features**				
Size of effusion (mm) (mean, SD) (number = 126)	36 (14)	36 (14)^*^	38 (13)^*^	29 (14) ^*^P <0.001
Tamponade (number, %) (number = 141)	94 (67)	50 (72)	32 (64)	12 (55)
**Routine pericardial fluid analyses**				
ADA IU/L (median, IQR) (number = 142)	51 (34 to 75)	59 (45 to 86)^*^	51 (34 to 77)^*^	17 (11 to 27) ^*^P <0.001
Total protein g/L (median, IQR) (number = 148)	60 (52 to 68)	59 (53 to 68)	63 (56 to 68)	56 (48 to 62)
Lactate dehydrogenase (median, IQR) (number = 135)	1419 (867 to 2305)	1553 (999 to 2800)^*^	1093 (725 to 1613)^*^	884 (442 to 2305) ^*^P = 0.02
Total PF WCC × 10^9^/l (Median, IQR) (number = 136)	2.1 (1.1 to 3.3)	2.0 (1.2 to 3.0)	2.2 (1.2 to 2.9)	3.0 (0.7 to 8.9)
Lymphocyte predominance (number/Number, %)^b^	52/107(49)	29/56 (52)^*^	22/34 (65)^*^	1/17 (6) ^*^P <0.001

^a^Nine patients refused testing or had unknown HIV status, and four HIV-infected patients had no CD4 cell count data. ^b^Multiple invalid results. ^*^P values indicate significant differences between patient groups. ADA, adenosine deaminase; ARV, anti-retroviral therapy; IQR, interquartile range; NYHA, New York Heart Association; PF, pericardial fluid; SD, standard deviation; WCC, white cell count.

To compare diagnostic accuracy between diagnostic tests and basic clinical predictors, given the demographic and clinical differences, a multivariate logistic regression model was developed to generate a quantitative estimate for the predictive value of clinical findings. Additional file 1: Table S1 in the online supplementary materials shows the results of the univariate and multivariate analyses. A set of the following basic clinical predictors: age ≤50 years, HIV-infection and the presence of night sweats offered the best predictive utility for TBP. Table 2 compares the diagnostic accuracy measures for the previously reported Tygerberg diagnostic index score ≥6 and the quantified clinical predictors of this cohort, using both a ROC-selected rule-in cut-point of >6.1 and Youden’s rule-out cut-pointof >3.5.

**Table 2:** Diagnostic accuracy measures of Xpert MTB/RIF and the biomarkers uIFNγ and ADA using ROC-selected cut-points (definite-TB for sensitivity and non-TB for specificity calculations)

**Diagnostic test**^**a**^	**Sensitivity (95% CI) (n/N)**^**c**^	**Specificity (95% CI) (n/N)**^**c**^	**Positive likelihood ratio, LR + (95% CI)**	**Negative likelihood ratio, LR- (95% CI)**	**Positive predictive value, PPV (95% CI)**	**Negative predictive value, NPV (95%CI)**
**Xpert MTB/RIF**	63.8% (52.4 to 75.1)	100% (85.6 to 100)	n/c	0.36	100%	86.6%
	44/69^* *1 *2^	^26/26 *4 *5^		(0.33 to 0.39)	(98.0 to 100)	(84.0 to 88.7)
**uIFNγ (Intergam) (Youden’s, rule-in and rule-out cut-points: ≥44 pg/ml)**^**c**^	95.7% (88.1 to 98.5)	96.3% (81.7 to 99.3)	25.8	0.045	91.7%	98.1%
	67/70^* *3^*P* <0.001	26/27^*6^	(3.6 to 184)	(0.023 to 0.09)	(88.1 to 94.3)	(96.8 to 98.9)
**ADA (rule-in cut-point: >107 IU/ml)**	15.7%	96%				
	(9.0 to 26.0)	(80.5 to 99.3)	3.93	0.88	62.7%	72.7%
	11/70	24/25	(0.21 to 72.5)	(0.85 to 0.91)	(51.4 to 72.8)	(69.7 to 75.4)
	^*1^*P* <0.001					
**ADA (cut-point in current clinical use: ≥35 IU/ml)**[5]	95.7%	84%				
	(88.1 to 98.5)	(65.4 to 93.6)	6.0	0.051	71.9%	97.9%
	67/70	21/25	(3.7 to 9.8)	(0.026 to 0.10)	(67.3 to 76.1)	(96.4 to 98.7)
	^*1 *3^*P* < 0.001	^*4^*P* = 0.03				
**Tygerberg score ≥6**	85.3%	77.3%				
	(75.9 to 81)	(56.6 to 89.9)	3.75	0.19	61.7%	92.5%
	58/68	17/22	(2.52 to 5.59)	(0.15 to 0.24)	(56.9 to 66.2)	(90.0 to 94.3)
	^*2^*P* = 0.004 ^*3^*P* = 0.04	^*5^*P* = 0.01 ^*6^*P* =0.04				
**Clinical predictors (rule-in cut-point: >6.1)**	60.8%	96.3%				
	(49.4 to 71.1)	(81.7 to 99.3)	16.4	0.41	87.6%	85.1%
	45/74	26/27	(2.25 to 119.9)	(0.38 to 0.44)	(82.4 to 91.4)	(82.5 to 87.5)
	^*3^*P* <0.001					
**Clinical predictors (Youden’s and rule-out cut-point: >3.5)**^**b**^	91.9%	81.5%	4.96	0.10	68.0%	95.9%
	(83.4 to 96.2)	(63.3 to 91.8)	(3.34 to 7.36)	(0.07 to 0.14)	(63.3 to 72.4)	(94 to 97.2)
	68/74	22/27				
	^*2^*P* <0.001	^*5^*P* = 0.02				
**Xpert MTB combined with uIFNγ (with uIFNγ if Xpert MTB/RIF negative)**	97.1%	100%	n/c			
	(89.9 to 99.2)	(86.7 to 100)		0.03	100%	98.8%
	66/68	25/25		(0.01 to 0.08)	(98.7 to 100)	(97.7 to 99.4)
**Xpert MTB combined with ADA (with ADA if Xpert MTB/RIF negative)**	98.4%	100%	n/c	0.02	100%	99.3%
	(91.7 to 99.7)	(85.7 to 100)		(0.002 to 0.11)	(98.7 to 100)	(98.4 to 99.7)
	63/64	23/23				

^a^Denominators other than 74 definite- and 27 non-TB patients indicate missing test results. Thus, 5/74 definite- and 1/27 non-TB patients did not have an Xpert MTB/RIF performed; 4/74 definite-TB did not have a uIFNγ (QFT-kit) test performed and 4/74 definite- and 2/27 non-TB did not have an ADA level measurement. Diagnostic accuracy measures presented in this table are for Xpert MTB/RIF performed on 1 ml unconcentrated PF, uIFNγ (both the QFT-kit and Intergam-kits) performed on the supernatants on 3 to 20 ml of centrifuged PF, and ADA performed on 1 to 8 ml of unprocessed PF.

^b^Youden’s index (the cut-point allowing the most correctly classified) and optimal ‘rule-out’ (the cut-point with the lowest LR-) cut-points for the uIFNγ (both the QFT-kit and Intergam-kits) were the same (QFT: 0.18 IU/l, Intergam: 44 pg/ml) respectively. ^c^*P*-values compare diagnostic tests for the indicated diagnostic accuracy measure, for example, sensitivity (specific proportions compared indicated by * and corresponding number symbol).

ADA: adenosine deaminase; CI: confidence interval; n/c: not calculable as denominator equals zero; PF, pericardial fluid; uIFNγ: unstimulated interferon gamma. The reference standard was culture and/or histopathology pathognomonic of TB^†^. TB prevalence = 30% used for predictive value estimates.

### Xpert MTB/RIF

**Figure 2:**
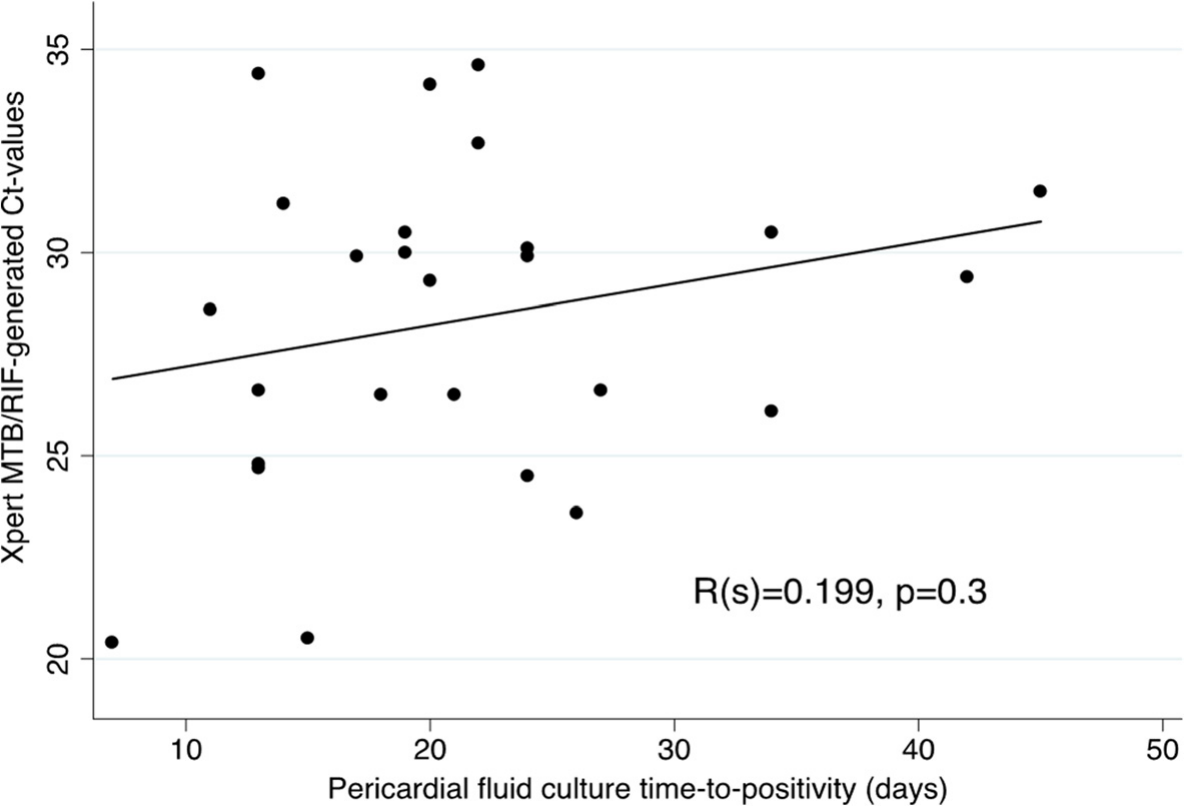
Scatter plot correlating pericardial fluid Xpert MTB/RIF-generated cycle threshold (CT) values with pericardial fluid liquid culture time-to-positivity (days).

Of the 151 patients who underwent pericardiocentesis, 9% (13/151) were not tested by the Xpert MTB/RIF assay because of missing samples. The range of PF volume used for a concentrated Xpert MTB/RIF was 3 to 20 ml. Spiking experiments in PF demonstrated that the Xpert MTB/RIF assay was detected in 100% of samples spiked with ≥75 CFUs/ml of PF [see Additional file 1: Figure S1, 4/4 replicates detected for 75, 100 and 150 CFUs/ml]. Table 3 shows that, overall, when comparing matched samples, the concentration of PF did not significantly increase the number of positive Xpert MTB/RIF results (1 ml unconcentrated: 41% (48/117) versus 20 ml concentrated 53% (62/117), *P* = 0.07). However, a higher number of indeterminate Xpert MTB/RIF results occurred when using concentrated versus unconcentrated (1 ml) PF samples (10.4% (14/134) versus 2.1% (3/138), *P* = 0.005). No difference in the mean C_T-_value ± SD Xpert MTB/RIF Spore (*Bacillus globigii*) positive control C_T-_values were noted between concentrated versus unconcentrated PF samples (26.7 ± 2.4 versus 26.4 ± 2.2, *P* = 0.4). No association between Xpert MTB/RIF-generated C_T_-values and PF liquid culture time-to-positivity (in days) was detected (R_s_ = 0.199, *P* = 0.3, Figure 2).

**Table 3:** Comparison of Xpert MTB/RIF assay test characteristics and diagnostic accuracy when using unconcentrated and concentrated pericardial fluid samples

**Pericardial fluid type**	**Indeterminate rate (number/total number of assays performed)**	**Internal positive control**^**a **^**C**_**T**_**-value (median, IQR)**	**Xpert MTB/RIF positive tests in definite and probable-TB patients**
Unconcentrated (1 ml)	2.1% (0.3 to 4.6) 3/138	26.2 (25.1 to 27.4)	41.0% (32.1 to 49.9) 48/117
Concentrated/centrifuged pellet (20 ml)	10.4% (5.3 to 15.6) 14/134	26.2 (25.1 to 27.6)	52.9% (43.9 to 62.0) 62/117
*P*-value^b^	0.005	0.9	0.07

A median of 20 ml of pericardial fluid was centrifuged at 3,000 × g for 15 minutes with the pellet re-suspended in sterile PBS, followed by Xpert MTB. ^a^Internal positive control: to evaluate potential PCR inhibition an unrelated fragment of fungal DNA (*Bacillus globigii*) is co-amplified in the reaction; ^b^*P*-values compare unconcentrated and concentrated pericardial fluid samples for particular Xpert. CI, confidence interval; IQR: Interquartile range; TB, tuberculosis,

Overall, the sensitivity (95% CI) of PF Xpert MTB/RIF was 63.8% (52.4 to 75.1) (Table 2). The sensitivity was higher in HIV-positive compared to HIV-negative patients (74.6% (61.7 to 84.2) versus 21.4% (7.6 to 47.6), *P* <0.001; see Additional file 1: Table S2), corresponding to higher bacillary loads in the PFs of HIV-positive patients (median (IQR) time-to-positivity (days) of liquid TB culture samples HIV-positive: 21 (17 to 29) versus HIV-negative: 25 (12 to 38), *P* <0.001]. Sensitivity did not decrease significantly when definite- and probable-TB patients were combined (*P* = 0.09, see Additional file 1: Table S3). Overall, Xpert MTB/RIF specificity (95% CI) was 100% (85.6 to 100) when using the composite reference standard, but only 69.0% (59.2 to 78.7) when the microbiological reference was used (*P* <0.001, see Additional file 1: Table S4). All positive PF Xpert MTB/RIF were rifampicin-sensitive giving a specificity (95% CI) for rifampicin resistance of 100% (88 to 100). Sensitivity could not be calculated.

### uIFNγ and ADA

**Figure 3:**
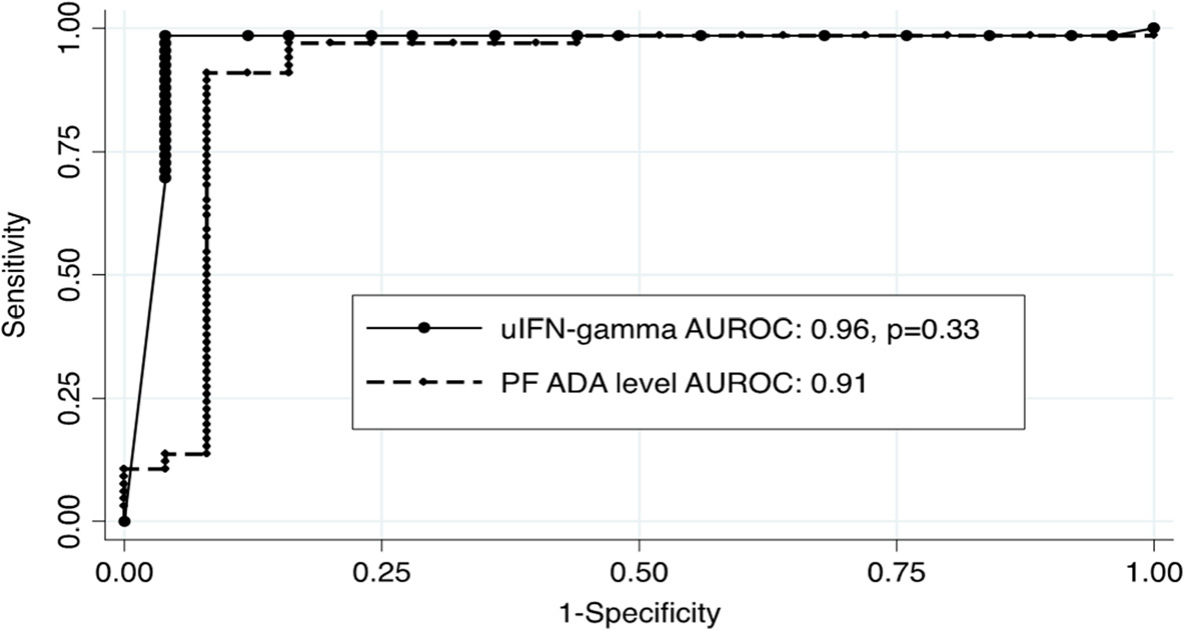
**Comparison of receiver operator characteristics (ROC) curves for the pericardial fluid biomarkers unstimulated interferon gamma (uIFN-gamma) and adenosine deaminase (ADA).** AUROC, area under the receiver operator characteristics curve. The point sensitivity versus specificity for the Xpert MTB/RIF is indicated on the graph as a solid black triangle.

Of the 151 patients enrolled, 6.6% (10/151) and 5.9% (9/151) were not subjected to uIFNγ and ADA assays, respectively. The optimal cut-points for uIFNγ and ADA levels to maximise diagnostic accuracy were determined using the ROC-curve shown in Figure 3. uIFNγ and ADA had similar areas under the ROC-curve (AUROC uIFNγ: 0.96 versus AUROC ADA: 0.91, *P* = 0.33). For uIFNγ, both the Youden’s index (cut-point that correctly classifies the most number of patients) and optimal rule-out cut-point was 44 pg/ml, while for ADA the cut-point currently utilised in routine clinical practice is >35 IU/l [22] compared to a ROC selected optimal rule-in cut-point which is 107 IU/L. These cut-points are used for the diagnostic accuracy analyses presented in Table 2.

The overall sensitivity (95% CI) of uIFNγ was 95.7% (88.1 to 98.5), which was similar to ADA using the clinical cut-point (Table 2). However, the specificity (95% CI) of uIFNγ was 96.3% (81.7 to 99.3) versus only 84% (65.4 to 93.6) for ADA at the clinical cut-point (*P* = 0.1). Similarly, although the sensitivity of the biomarkers uIFNγ and ADA was similar for both HIV-positive and -negative patients, the specificity of ADA (clinical cut-point) was lower in HIV-positive patients (*P* <0.001, Additional file 1: Table S2).

### Comparative diagnostic accuracy of routine and new same-day diagnostic tools

We further interrogated the potential clinical utility of routine (that is, ADA assay) and new same-day diagnostic tools (that is, uIFNγ and Xpert MTB/RIF) by comparing positive (LR+) and negative (LR-) likelihood ratios (Table 2) and positive (PPV) and negative (NPV) predictive values at different prevalence rates of TB (TB prevalence = 30% in Table 2, TB prevalence of 10%, 30%, and 50% presented in Additional file 1: Table S5). With 100% specificity, the LR + and PPV (irrespective of TB prevalence) for Xpert MTB/RIF was excellent, but sensitivity was suboptimal compared to other biomarkers and clinical predictors and thus LR- was only 0.49. Compared to ADA (clinical cut-point 35 IU/ml) and clinical predictors, the biomarker uIFNγ (cut-point 44 pg/ml) offers better rule-in utility with higher sensitivity, LR+, and in high TB prevalence settings (prevalence = 50%) a PPV of 96.9% (95.1 to 98.1) [see Additional file 1: Table S5]. Both ADA (clinical cut-point 35 IU/ml) and uIFNγ (cut-point 44 pg/ml) with sensitivities >95% offer excellent rule-out utility with low LR- and NPV just below 95% in high TB prevalence settings (prevalence = 50%, Additional file 1: Table S5).

### Xpert MTB/RIF in combination with pericardial fluid biomarkers

Table 2 shows the diagnostic accuracy of using PF Xpert MTB/RIF together with the biomarkers ADA and uIFNγ. Performing a PF Xpert MTB/RIF followed by either ADA or uIFNγ offered equivalent excellent diagnostic accuracy with sensitivity and specificities >97%.

## Discussion

The performance of the new WHO-endorsed, Xpert MTB/RIF assay has recently been reported for some types of extra-pulmonary TB such as TB lymphadenitis [23], pleural TB [24], and TB meningitis [25]. However, there are no comprehensive data about TBP to guide clinical practice. Here we report on the first large comprehensive study of Xpert MTB/RIF for the diagnosis of pericardial TB [5,10]. It is also the first study to compare Xpert MTB/RIF to several alternative diagnostic assays, including ADA and IFN-γ, and to evaluate test performance outcomes in a TB and HIV-endemic setting.

The key findings of our study are that: (1) uIFNγ offers superior accuracy for the diagnosis of microbiologically confirmed TBP compared to the new Xpert MTB/RIF test and the established ADA assay; (2) PF Xpert MTB/RIF could bacteriologically confirm a TB diagnosis (and allow for drug susceptibility testing) in two thirds of patients with suspected TBP; (3) PF uIFNγ offered better rule-in diagnostic utility compared to ADA in current clinical use, while both tests could rapidly rule-out TBP; (4) PF Xpert MTB/RIF, when combined with either ADA or uIFNγ, offers >97% sensitivity and specificity for TBP diagnosis; and (5) concentration of PF samples prior to Xpert MTB/RIF testing increased the number of ‘indeterminate’ tests without significantly improving diagnostic yield.

Xpert MTB/RIF testing is undergoing phased implementation in a number of high burden settings for routine diagnosis of pulmonary TB [26,27]. There is limited information on the diagnostic utility of the test in extrapulmonary cases of TB, and, in particular, Xpert MTB/RIF performance has only been evaluated in a very small number of PF samples [13]. Our study is the largest systematic evaluation to date, and the first to examine Xpert MTB/RIF level of detection in PF and explore the effects of concentrating larger volumes of PF on Xpert MTB/RIF performance. Importantly, Xpert MTB/RIF testing could microbiologically confirm TB and allow drug susceptibility testing in almost two thirds of culture-positive cases, which is higher than in other body cavity fluids, including pleural, non-sputum biological fluids such as urine, and similar to performance in induced sputum specimens [13,28,29]. Preliminary level of detection experiments suggest that the Xpert MTB/RIF assay could reliably detect PF samples spiked with ≥75 cfus/ml of H37Rv, which is lower than the 131 cfu/ml limit of detection found in spiked sputum samples [30]. Further studies with more replicates are required to confirm this finding. However, the diagnostic yield from PF was not improved by centrifugation of larger volumes and concentration only increased the number of ‘indeterminate’ test results, although this was not the result of an increase in PCR inhibition. The increased error rate may have resulted from reaction failure secondary to large amounts of pelleted blood and other inflammatory proteins found in pericardial exudates. Methods to further digest these proteins or the addition of a PCR-friendly blood lysis buffer may help to decrease error rates [31,32]. Interestingly, unlike in sputum and pleural samples, no correlation was found between Xpert MTB/RIF-generated C_T_-values and liquid culture time-to-positivity using PF [33]. However, the sensitivity of Xpert MTB/RIF was found to be significantly higher in HIV-positive versus negative patients, and this was due to the higher bacillary loads, as measure by liquid culture time-to-positivity (TTP), found in the PF of HIV-positive versus -negative TBP. This sensitivity difference may impact on the utility of Xpert MTB/RIF in low HIV prevalence settings.

Proof-of-principle studies in TB pericarditis have demonstrated the potential utility of using uIFNγ PF levels for diagnosis of TB pericarditis [6,14,34]. Although it can be easily measured, it is not routinely performed due to its high cost and the kits only being available in a 96-well format, which would lead to a considerable wastage of unused wells [5,35]. However, the recent availability of a low-cost assay (Intergam, Antrum Biotech, Cape Town, South Africa) which is tested in this study may allow for more widespread use of uIFNγ for the diagnosis of TBP in clinical practice. In this study, using ROC-curve analysis, we demonstrate an optimal cut-point of 44 pg/ml, and show that with this cut-point of uIFNγ we could detect almost all definite-TB cases (missing only three cases) and incorrectly classified only one non-TB case.

Are the findings of this study generalisable to other settings, and does either Xpert MTB/RIF or uIFNγ testing potentially offer utility beyond existing same-day diagnostic tools, such as smear microscopy, PF ADA measurements and/or basic clinical information? In this study we compare the utility of Xpert MTB/RIF, uIFNγ or ADA, alone or in combination across different TB prevalence rates, focusing on the diagnostic priorities of rapid rule-in and rule-out, as well as bacteriologically confirmed diagnosis. In a high prevalence setting (TB prevalence >30%), Xpert MTB/RIF and uIFNγ outperforms ADA and basic clinical predictors for rapid rule-in (highest LR + and PPV). However, both ADA and uIFNγ offer equivalent rapid rule-out utility, outperforming Xpert MTB/RIF and clinical predictors. Combining Xpert MTB/RIF testing followed by ADA or uIFNγ in Xpert-negative PF maximised both sensitivity and specificity to >97% for TBP diagnosis. This may offer the best diagnostic approach in high burden settings, especially where drug-susceptibility testing is desirable, but the cost of a two test algorithm will remain a key consideration in resource-poor conditions where TB is endemic. Xpert MTB/RIF currently costs approximately US$20/test, while ADA measurement is less than US$0.1/test. Intergam kits are not currently commercially available so the cost is unknown but likely to be only slightly more than smear microscopy. Prospective studies of the cost-effectiveness of diagnostic options are needed before it can be considered for clinical practice.

Our study had a number of important limitations. This study did not optimise PF sample volumes or processing beyond comparing two volumes and a simple centrifugation step thought applicable to resource-limited settings. The use of different volumes or alternative processing methods may have improved Xpert MTB/RIF sensitivity and/or decreased the high indeterminate rate found. A low number of replicates were performed in limit of detection experiments and these findings should be confirmed in further studies. The study was conducted in a high TB and HIV burden setting, which may limit the generalisability of the findings. Performance may differ in a low TB burden setting and where HIV co-infection rates and, hence, bacterial load, are lower, such as Europe and the US. However, the use of diagnostic accuracy measures that are less affected by prevalence, such as LRs, and generating estimates across varying TB prevalence rates helps to highlight potential performance differences between low and high burden settings and, hence, improve generalisability. Whilst this is the largest study that has comprehensively evaluated several diagnostic strategies and tools in the same prospective cohort, the sample size was limited in the non-TB group. The small number of non-TB patients reflects the high burden of infectious and HIV-related disease in the South African environment [27]. Although the use of a combined reference standard may introduce a minor degree of selection bias, this consideration is outweighed by the avoidance of misclassification bias when using a culture only reference (data provided in the online supplementary materials).

## Conclusions

In conclusion, uIFNγ offers superior accuracy for the diagnosis of microbiologically confirmed TBP compared to the new Xpert MTB/RIF test and the established ADA assay, performed using available Xpert MTB/RIF testing protocols without fluid-specific optimisation beyond simple centrifugation. These data suggest that the uIFNγ assay may be the optimal first line test for the diagnosis of TB pericarditis, and merits consideration for implementation in clinical practice. Furthermore, PF Xpert MTB/RIF, when combined with either ADA or uIFNγ, offers high sensitivity and specificity for TBP diagnosis. Studies are needed to test the utility and cost-effectiveness of a two-test strategy, which may be preferred in HIV-positive patients where biomarker specificity may be reduced. Collectively, these data suggest that a biomarker-oriented approach may be feasible and accurate for the diagnosis of suspected TBP in a high TB and HIV prevalence setting.

## Abbreviations

ADA: Adenosine deaminase; ARV: Anti-retroviral therapy; AUROC: Area under the receiver operator characteristics curve; CFU: Colony forming units; CI: Confidence interval; HIV: Human immunodeficiency virus; IQR: Interquartile range; LR-: Negative likelihood ratio; LR+: Positive likelihood ratio; *M. tb*: *Mycobacterium tuberculosis*; NPV: Negative predictive value; NYHA: New York Heart Association; PF: Pericardial fluid; PPV: Positive predictive value; SD: Standard deviation; TB: Tuberculosis; TBP: Tuberculous pericarditis; uIFNγ: Unstimulated interferon-gamma; WCC: White cell count.

## Competing interests

KD and UG have performed consultancy work for Antrum Biotech (Pty) Ltd, a University of Cape Town co-owned spin-off company, and kits for the study were donated by the company. However, Antrum Biotech played no role in study design, data analysis or its publication. The other authors declare that they have no competing interests.

## Authors’ contributions

SP, JGP, KD and BMM contributed to the conception and design of the study, the acquisition of data, analysis and interpretation of data, and drafting of the manuscript. ZK, RM, GT, UG, and MN contributed to the acquisition and interpretation of data and drafting of the manuscript. All authors read and approved the final version of the manuscript.

## Pre-publication history

The pre-publication history for this paper can be accessed here:

http://www.biomedcentral.com/1741-7015/12/101/prepub

## Supplementary Material

Additional file 1 Table S1 Table S2-5 and Figure S1. Click here for file

## References

[B1] World Health Organization Global Tuberculosis Report 2013 2013 Geneva

[B2] Maartens G Wilkinson RJ Tuberculosis Lancet 2007 370 2030 2043 17719083 10.1016/S0140-6736(07)61262-8

[B3] Mayosi BM Wiysonge CS Ntsekhe M Volmink JA Gumedze F Maartens G Aje A Thomas BM Thomas KM Awotedu AA Thembela B Mntla P Maritz F Blackett KN Nkouonlack DC Burch VC Rebe K Parrish A Sliwa K Vezi BZ Alam N Brown BG Gould T Visser T Magula NP Commerford PJ Mortality in patients treated for tuberculous pericarditis in sub-Saharan Africa S Afr Med J 2008 98 36 40 18270639

[B4] Damasceno A Mayosi BM Sani M Ogah OS Mondo C Dike O Dzudie A Kouam Kouam C Suliman A Schrueder N Yonga G Ba SA Maru F Alemayehu B Edwards C Davison BA Cotter G Sliwa K The causes, treatment, and outcome of acute heart failure in 1006 Africans from 9 countries: results of the sub-saharan africa survey of heart failure Arch Int Med 2012 172 1386 1394 22945249 10.1001/archinternmed.2012.3310

[B5] Mayosi BM Burgess LJ Doubell AF Tuberculous pericarditis Circulation 2005 112 3608 3616 16330703 10.1161/CIRCULATIONAHA.105.543066

[B6] Reuter H Burgess L van Vuuren W Doubell A Diagnosing tuberculous pericarditis QJM 2006 99 827 839 17121764 10.1093/qjmed/hcl123

[B7] Strang G Latouf S Commerford P Roditi D Duncan-Traill G Barlow D Forder A Bedside culture to confirm tuberculous pericarditis Lancet 1991 338 1600 1601 1684009 10.1016/0140-6736(91)92433-3

[B8] Holtz TH Kabera G Mthiyane T Zingoni T Nadesan S Ross D Allen J Chideya S Sunpath H Rustomjee R Use of a WHO-recommended algorithm to reduce mortality in seriously ill patients with HIV infection and smear-negative pulmonary tuberculosis in South Africa: an observational cohort study Lancet Infect Dis 2011 11 533 540 21514234 10.1016/S1473-3099(11)70057-3

[B9] Boehme CC Nabeta P Hillemann D Nicol MP Shenai S Krapp F Allen J Tahirli R Blakemore R Rustomjee R Milovic A Jones M O'Brien SM Persing DH Ruesch-Gerdes S Gotuzzo E Rodrigues C Alland D Perkins MD Rapid molecular detection of tuberculosis and rifampin resistance N Engl J Med 2010 363 1005 1015 20825313 10.1056/NEJMoa0907847 PMC2947799

[B10] Policy statement: automated real-time nucleic acid amplification technology for rapid and simultaneous detection of tuberculosis and rifampicin resistance Xpert mtb/rif system http://whqlibdoc.who.int/publications/2011/9789241501545_eng.pdf 26158191

[B11] Steingart KR Sohn H Schiller I Kloda LA Boehme CC Pai M Dendukuri N Xpert(R) MTB/RIF assay for pulmonary tuberculosis and rifampicin resistance in adults Cochrane Database Syst Rev 2013 1 CD009593 10.1002/14651858.CD009593.pub2 PMC4470352 23440842

[B12] Dhana AV Howell P Sanne I Spencer D Identification of Mycobacterium tuberculosis from pericardial fluid using the new Xpert MTB/RIF assay BMJ Case Rep 2013 doi:10.1136/bcr-2013-200615 10.1136/bcr-2013-200615 PMC3762401 23986128

[B13] Vadwai V Boehme C Nabeta P Shetty A Alland D Rodrigues C Xpert MTB/RIF: a new pillar in diagnosis of extrapulmonary tuberculosis? J Clin Microbiol 2011 49 2540 2545 21593262 10.1128/JCM.02319-10 PMC3147857

[B14] Burgess LJ Reuter H Carstens ME Taljaard JJ Doubell AF The use of adenosine deaminase and interferon-{gamma} as diagnostic tools for tuberculous pericarditis Chest 2002 122 900 905 12226030 10.1378/chest.122.3.900

[B15] Dheda K van Zyl-Smit RN Sechi LA Badri M Meldau R Meldau S Symons G Semple PL Maredza A Dawson R Wainwright H Whitelaw A Vallie Y Raubenheimer P Bateman ED Zumla A Utility of quantitative T-cell responses versus unstimulated interferon-{gamma} for the diagnosis of pleural tuberculosis Eur Respir J 2009 34 1118 1126 19386693 10.1183/09031936.00005309

[B16] Reuter H Burgess LJ Carstens ME Doubell AF Adenosine deaminase activity–more than a diagnostic tool in tuberculous pericarditis Cardiovasc J S Afr 2005 16 143 147 16049586

[B17] Mayosi BM Wiysonge CS Ntsekhe M Volmink JA Gumedze F Maartens G Aje A Thomas BM Thomas KM Awotedu AA Thembela B Mntla P Maritz F Blackett KN Nkouonlack DC Burch VC Rebe K Parish A Sliwa K Vezi BZ Alam N Brown BG Gould T Visser T Shey MS Magula NP Commerford PJ Clinical characteristics and initial management of patients with tuberculous pericarditis in the HIV era: the Investigation of the Management of Pericarditis in Africa (IMPI Africa) registry BMC Infect Dis 2006 6 2 16396690 10.1186/1471-2334-6-2 PMC1352368

[B18] Adenosine Deaminase Assay [http://www.diazyme.com/adenosine-deaminase-ada]

[B19] White IR Royston P Wood AM Multiple imputation using chained equations: issues and guidance for practice Stat Med 2011 30 377 399 21225900 10.1002/sim.4067

[B20] Schisterman EF Perkins NJ Liu A Bondell H Optimal cut-point and its corresponding Youden Index to discriminate individuals using pooled blood samples Epidemiology 2005 16 73 81 15613948 10.1097/01.ede.0000147512.81966.ba

[B21] Bossuyt PM Reitsma JB Bruns DE Gatsonis CA Glasziou PP Irwig LM Moher D Rennie D de Vet HC Lijmer JG The STARD statement for reporting studies of diagnostic accuracy: explanation and elaboration Ann Intern Med 2003 138 W1 W12 12513067 10.7326/0003-4819-138-1-200301070-00012-w1

[B22] Burgess LJ Reuter H Carstens ME Taljaard JJ Doubell AF The use of adenosine deaminase and interferon-gamma as diagnostic tools for tuberculous pericarditis Chest 2002 122 900 905 12226030 10.1378/chest.122.3.900

[B23] Ligthelm LJ Nicol MP Hoek KG Jacobson R van Helden PD Marais BJ Warren RM Wright CA Xpert MTB/RIF for rapid diagnosis of tuberculous lymphadenitis from fine-needle-aspiration biopsy specimens J Clin Microbiol 2011 49 3967 3970 21880965 10.1128/JCM.01310-11 PMC3209093

[B24] Meldau R Peter J Theron G Calligaro G Allwood B Symons G Khalfey H Ntombenhle G Govender U Binder A van Zyl-Smit R Dheda K Comparison of same day diagnostic tools including Gene Xpert and unstimulated IFN-gamma for the evaluation of pleural tuberculosis: a prospective cohort study BMC Pulm Med 2014 14 58 24708530 10.1186/1471-2466-14-58 PMC4108018

[B25] Patel VB Theron G Lenders L Matinyena B Connolly C Singh R Coovadia Y Ndung’u T Dheda K Diagnostic accuracy of quantitative PCR (Xpert MTB/RIF) for tuberculous meningitis in a high burden setting: a prospective study PLoS Med 2013 10 e1001536 24167451 10.1371/journal.pmed.1001536 PMC3805498

[B26] Lawn SD Mwaba P Bates M Piatek A Alexander H Marais BJ Cuevas LE McHugh TD Zijenah L Kapata N Abubakar I McNerney R Hoelscher M Memish ZA Migliori GB Kim P Maeurer M Schito M Zumla A Advances in tuberculosis diagnostics: the Xpert MTB/RIF assay and future prospects for a point-of-care test Lancet Infect Dis 2013 13 349 361 23531388 10.1016/S1473-3099(13)70008-2 PMC4844338

[B27] Mayosi BM Lawn JE van Niekerk A Bradshaw D Abdool Karim SS Coovadia HM Health in South Africa: changes and challenges since 2009 Lancet 2012 380 2029 2043 23201214 10.1016/S0140-6736(12)61814-5

[B28] Peter JG Theron G Muchinga TE Govender U Dheda K The diagnostic accuracy of urine-based Xpert MTB/RIF in HIV-infected hospitalized patients who are smear-negative or sputum scarce PLoS One 2012 7 e39966 22815718 10.1371/journal.pone.0039966 PMC3392260

[B29] Peter JG Theron G Pooran A Thomas J Pascoe M Dheda K Comparison of two methods for acquisition of sputum samples for diagnosis of suspected tuberculosis in smear-negative or sputum-scarce people: a randomised controlled trial Lancet Respir Med 2013 1 471 478 24429245 10.1016/S2213-2600(13)70120-6 PMC4632198

[B30] Helb D Jones M Story E Boehme C Wallace E Ho K Kop J Owens MR Rodgers R Banada P Safi H Blakemore R Lan NT Jones-López EC Levi M Burday M Ayakaka I Mugerwa RD McMillan B Winn-Deen E Christel L Dailey P Perkins MD Persing DH Alland D Rapid detection of Mycobacterium tuberculosis and rifampin resistance by use of on-demand, near-patient technology J Clin Microbiol 2010 48 229 237 19864480 10.1128/JCM.01463-09 PMC2812290

[B31] Hillemann D Rusch-Gerdes S Boehme C Richter E Rapid molecular detection of extrapulmonary tuberculosis by the automated GeneXpert MTB/RIF system J Clin Microbiol 2011 49 1202 1205 21270230 10.1128/JCM.02268-10 PMC3122824

[B32] Banada PP Koshy R Alland D Detection of Mycobacterium tuberculosis in blood by use of the Xpert MTB/RIF assay J Clin Microbiol 2013 51 2317 2322 23678063 10.1128/JCM.00332-13 PMC3697682

[B33] Theron G Peter J van Zyl-Smit R Mishra H Streicher E Murray S Dawson R Whitelaw A Hoelscher M Sharma S Pai M Warren R Dheda K Evaluation of the Xpert MTB/RIF assay for the diagnosis of pulmonary tuberculosis in a high HIV prevalence setting Am J Respir Crit Care Med 2011 184 132 140 21493734 10.1164/rccm.201101-0056OC

[B34] Latouf SE Ress SR Lukey PT Commerford PJ Interferon-gamma in pericardial aspirates: a new, sensitive and specific test for the diagnosis of tuberculous pericarditis Circulation 1991 84 II 149 [Abstract]

[B35] Sharma SK Banga A Pleural fluid interferon-gamma and adenosine deaminase levels in tuberculosis pleural effusion: a cost-effectiveness analysis J Clin Lab Anal 2005 19 40 46 15756707 10.1002/jcla.20054 PMC6808038

