# Supplementary material for: Diagnostic accuracy of quantitative PCR (Xpert MTB/RIF) for tuberculous pericarditis compared to adenosine deaminase and unstimulated interferon-γ in a high burden setting: a prospective study
Source: BMC Med. 2014 Jun 18;12:101. doi: 10.1186/1741-7015-12-101 (PMC4073812; doi:10.1186/1741-7015-12-101)
Supplement: Additional file 1 — Table S1 Table S2-5 and Figure S1. [file 1741-7015-12-101-S1.docx]

**Diagnostic Accuracy of Quantitative PCR (Xpert MTB/RIF) for Tuberculous Pericarditis Compared to Adenosine Deaminase and Unstimulated Interferon-γ Assays in a High Burden Setting: A Prospective Study**

Shaheen Pandie, MBChB, MMed (Med);^1,*^ Jonathan G. Peter, MBChB, MMed (Med);^2,3,*^ Zita S. Kerbelker, MBChB;^1^ Richard Meldau, MSc;^2^ Grant Theron, PhD;^2^ Ureshnie Govender,PhD;^2^ Mpiko Ntsekhe, MD, PhD;^1^ Keertan Dheda, MBBCh, PhD;^2, 4, #^ Bongani M. Mayosi, MBChB, DPhil ^1,4, #^

^1^The Cardiac Clinic, Department of Medicine, Groote Schuur Hospital and University of Cape Town, Cape Town, South Africa; ^2^ Lung Infection and Immunity Unit, Division of Pulmonology and UCT Lung Institute, Department of Medicine, Groote Schuur Hospital and University of Cape Town, Cape Town, South Africa; ^3^TB Vaccine Group, Jenner Institute, University of Oxford, Oxford, United Kingdom; ^4^Institute of Infectious Diseases and Molecular Medicine, University of Cape Town, Cape Town, South Africa.

*^,#^ These authors contributed equally to the work

Corresponding author: Dr. Bongani M. Mayosi

Department of Medicine, Old Groote Schuur Hospital, Groote Schuur Drive, Observatory, 7925, Cape Town, South Africa

Phone: +27 (0)21 406-6200 (0ffice); Fax: +27 (0)21 448-6815; Cell: +27 82 372 7655

Email: [Bongani.Mayosi@uct.ac.za](mailto:Bongani.Mayosi@uct.ac.za)

**Supplementary methods
Study population**

Between October 2009 and September 2012 any patient with suspected TB pericarditis referred for enrolment in the Investigation of Management of Pericarditis (IMPI) registry, was screened for potential inclusion. Participants were referred from five hospitals (four district-level hospitals: New Somerset Hospital, Victoria Hospital, GF Jooste Hospital, and Karl Bremmer Hospital); and one tertiary-level hospital, Groote Schuur, in Cape Town, South Africa. Inclusion criteria included: (1) the presence of a large pericardial effusion amenable to safe pericardiocentesis (greater than 10mm echo-free space around the heart demonstrated on echocardiogram or ultrasound); (2) age 18 years or older; and (3) the provision of informed consent. Exclusion criteria included (1) positive urinary pregnancy test; (2) anti-TB treatment initiated >1 week prior to pericardiocentesis; and (3) refusal or inability to sign consent. The study was approved by the University of Cape Town Human Research Committee, as an official IMPI sub-study.

**Diagnostic Sample Collection and Handling**

The attending clinicians at referral hospital sites were responsible for the initial TB diagnostic work-up (including chest x-ray [CXR]; the collection of expectorated sputum; and/or other samples for diagnostic testing [Table S7]) and for the treatment decisions of enrolled participants. Eligible participants were identified and referred for diagnostic and/or therapeutic pericardiocentesis and concomitantly screened for study inclusion. Pericardiocentesis was performed by proficient physicians in keeping with standard safety and monitoring precautions. A minimum of 60ml of pericardial fluid **(**PF) was collected for diagnostic testing. If sufficient fluid was aspirated, four sodium heparin, two Ethylenediaminetetraacetic acid (EDTA); and two clear tubes where collected. PF was sent to the National Health Laboratory Services (NHLS) for measurement of ADA and lactate dehydrogenase (LDH) levels, cell counts and cytology, as well as routine TB diagnostics consisting of concentrated fluorescence smear microscopy and MGIT liquid culture (MGIT 960, BD Diagnostics, USA). Drug susceptibility testing was performed on positive culture isolates using the Genotype MDRTBplus assay (Hain, Lifesciences). In addition, aliquots of PF were processed as follows: (1) four 2 ml aliquots in EDTA tubes (unconcentrated), (2) five 2ml aliquots in sodium heparin tubes (unconcentrated), (3) four 2 ml aliquots in sodium heparin tubes (concentrated), (4) four 2 ml aliquots in plain tubes (concentrated), and (5) banking of remaining 5ml of PF. PF was stored at -20 °C, for later measurement of uIFNγ levels and performance of the Xpert MTB/RIF assay. Investigators performing Xpert MTB/RIF and uIFNγ were blinded to clinical and routine tuberculous diagnostic findings and final diagnostic classification.

**Diagnostic classification**

All patients that were included had a clinical diagnosis of a pericarditis or a pericardial syndrome, with echocardiographic confirmation of a pericardial effusion. Pericardial syndrome was defined as:

1. Clinical features of pericarditis including chest pain and shortness of breath
2. Clinical features of heart failure in the setting of a confirmed pericardial effusion
3. Compatible ECG and or echocardiographic findings of a pericarditis and / or pericardial effusion

Patients were categorised into the following diagnostic groups based on a combination of pericardial and non-pericardial sample culture results, histopathology of pericardial biopsy samples, basic pericardial fluid characteristics, and the commencement of TB treatment as follows:

1. Definite-TB: At least 1 *M. tb* sample positive by liquid culture (either pericardial or non-pericardial) and/or granulomatous inflammation on pericardial tissue histology (i.e., composite reference standard). Non-pericardial samples included sputum, lymph node biopsy, pleural fluid, blood, cerebrospinal fluid and blood.
2. Probable-TB: Clinical (Tygerberg TB Pericarditis Diagnostic Index Score (TDIS) of ≥6) and / or biochemical diagnosis characterised by elevated ADA and lymphocytic predominance; not meeting the criterion for definite-TB, but given TB treatment. The TDIS is a weighted score comprising clinical variables (weight loss, night sweats and fever) and blood biochemistry (serum globulin >40g/l and leukocyte count <10x10^9^/l).
3. Non-TB: No microbiological evidence of *M. tb* and an alternative diagnosis is available.

**Modeling clinical predictors using multiple imputation**

A univariate analysis was used to determined basic clinical predictors of definite-TB pericarditis. A set of multivariate clinical predictors was generated using logistic regression modeling. Multiple imputation by chained Equations was used to impute missing data prior to model building [^1^](#_ENREF_1). The variables included in the logistic regression modeling included the following (number of missing data points for each variable that were imputated is indicated in the brackets): sex (0), age (1), previous TB history (5), hivstatus (9), CD4 cell count (4), Receiving HAART (7), cough ≥ 2 weeks (6), self-reported weight loss (6), recent fever (5), night sweats (6), shortness of breath (5), chest pain (7), measured weight (42), NYHA grade (I-II, III-IV) (17), temperature (6), pulse rate (5), Systolic and diastolic blood pressure (6), presence of pulsus paradoxus (13), and an elevated Jugular venous pressure (7). Using ROC analysis the continous variable age was dichotomised to maximise discriminatory utility to generate a binary variable (age>40years). Different data appeared to be missing for different patients in a random fashion. Rounded ß-coefficients from the reduced model of significant variables were used to generate scores to quantitate relevant clinical predictors included in the final model. ROC analysis was performed and three cut-points were selected for rule-in, Youden’s index (the optimal mathematical balance between sensitivity and specificity [^2^](#_ENREF_2)) and rule-out value. Diagnostic accuracy, including 95% confidence intervals, for each cut-point was assessed using sensitivity, specificity, positive and negative predictive values (PPV, NPV) and positive likelihood ratio (LR+). STATA IC, version 11 (Stata Corp, Texas, USA) was used for all statistical analyses.

**Supplementary Results - Tables**

**1. Logistic regression to examine basic clinical predictors of TBP and development of quantitative estimate of basic clinical findings for comparison to novel and existing same-day diagnostic tools**

Table S1. Univariate and multivariate analyses for basic clinical associates of TB pericarditis

| **Patient characteristic** | **Univariate analysis** | | |
| --- | --- | --- | --- |
|  | OR  (95% CI) | P-value | β-coefficient |
| Age (continuous) | 0.08 (0.02-0.26) | <0.001 | n/a |
| Age≤50 years^†^ (dichotomised) | 12.0 (4.0-35.5) | <0.001 | n/a |
| Previous TB | 5.5 (1.2-25.7) | 0.03 | n/a |
| HIV-positive | 9.0 (2.9-28.2) | <0.001 | n/a |
| Self-reported fevers | 3.7 (1.4-9.7) | 0.008 | n/a |
| Night sweats | 19.3 (5.3-71.0) | <0.001 | n/a |
| NYHA grade I-II | 5.6 (1.6-18.8) | 0.006 | n/a |
| **Multivariate analysis (clinical and radiology predictors only)** | | | |
| Age ≤ 50 years | 5.8 (1.3-25.5) | 0.02 | 1.75 |
| HIV-positive | 14.3 (2.6-77.5) | 0.002 | 2.66 |
| Night sweats | 32.4 (5.3-197.5) | <0.001 | 3.48 |

^†^Receiver operating characteristic (ROC) curve-selected cut-point maximizing discriminatory utility used to dichotomise the continuous variable “age”

OR: odds ratio; TB: Tuberculosis; NYHA: New York Heart Association dyspnea grade

**2. Diagnostic test performance stratified by HIV status in primary analysis**

Table S2. Diagnostic accuracy measures of Xpert MTB/RIF and the biomarkers uIFNγ and ADA using ROC-selected cut-points and stratified by HIV status (definite-TB for sensitivity and non-TB for specificity calculations). The reference standard was culture and/ or histopathology pathognomonic of TB^†^.

|  | **Sensitivity**  **(95% CI)**  **(n/N)** | **Specificity**  **(95% CI)**  **(n/N)** | **Positive Likelihood ratio, LR+ (95% CI)** | **Negative Likelihood ratio, LR- (95% CI)** |
| --- | --- | --- | --- | --- |
| **HIV-positive** |  | | | |
| Xpert MTB/RIF | 74.6%  (61.7-84.2)  (41/55)^*^ | 100%  (56.6-100)  (5/5) | n/c | 0.25 (0.221-0.293) |
| uIFNγ (QFT-kit) (Cut-point: ≥0.18 IU/l)^‡^ | 98.2% (90.6-99.7) 55/56 | 80% (37.6-96.4) 4/5 | 4.9 (0.7-34.9) | 0.02 (0.003-0.179) |
| ADA (Clinical cut-point: ≥35)^‡^ | 96.4% (87.7-99.0) 53/55 | 40% (11.8-76.9) 2/5^*^ | 1.6 (0.8-3.1) | 0.09 (0.007-1.05) |
| **HIV-negative** |  | | | |
| Xpert MTB/RIF | 21.4%  (7.6-47.6)  (3/14) ^*^p<0.001 | 100%  (7.2-100)  (13/13) | n/c | 0.79 (0.658-0.939) |
| uIFNγ (QFT-kit) (Cut-point: ≥0.18 IU/l) | 100% (78.5-100) 14/14 | 100% (77.2-100) 13/13 | n/c | n/c |
| ADA (Clinical cut-point: ≥35) | 93.3% (70.2-98.8) 14/15 | 100% (75.8-100) 12/12 ^*^p<0.001 | n/c | 0.067 (0.009-0.473) |

^*^p-values compare HIV-positive and negative groups for a specific diagnostic test e.g Xpert MTB/RIF and accuracy measure e.g. sensitivity. P>0.05 are not shown.

^†^Nine definite- and non-TB patients had an unknown HIV status. Thus, denominators other than 59 definite- and 5 non-TB HIV-positive patients and 15 definite- and 13 non-TB HIV-negative patients indicate missing test results.

^‡^Youden’s index is the cut-point allowing the most correctly classified patients

uIFNγ: unstimualted interferon gamma; ADA: adenosine deaminase; CI: Confidence interval; n/c: not calculatable

**3. Alternative analyses for improved methodological validity in the context of the need to utilise a composite diagnostic reference standard.**

**i) Analysis of diagnostic accuracy using definite and probable TB groups combined for sensitivity calculations**

Table S3. Diagnostic accuracy measures of Xpert MTB/RIF and the biomarkers uIFNγ and ADA using ROC-selected cut-points (definite- and probable-TB groups combined for sensitivity and non-TB for specificity calculations). The reference standard was culture and/ or histopathology pathognomonic of TB^†^.

|  | **Sensitivity**  **(95% CI)**  **(n/N)** | **Specificity**  **(95% CI)**  **(n/N)** | **Positive Likelihood ratio, LR+ (95% CI)** | **Negative Likelihood ratio, LR- (95% CI)** |
| --- | --- | --- | --- | --- |
| **Xpert MTB/RIF** | 50.9%  (41.8-60.0)  (57/112)^*^ | 100%  (85.6-100)  (26/26) | n/c | 0.491  (0.474-0.509) |
| **uIFNγ (QFT-kit) (Cut-point: ≥0.18 IU/l)^‡^** | 95.6%  (90.1-98.1) 109/114 ^*^p<0.001 | 96.3%  (81.7-99.3) 26/27^*^ | 25.8  (3.6-183.4) | 0.046  (0.031-0.068) |
| **uIFNγ (Intergam) (Cut-point: 44pg/ml) ^‡^** | 92.1%  (85.7-95.7) 105/114 | 96.3%  (81.7-99.3) 26/27 | 24.9  (3.5-176) | 0.082  (0.066-0.10) |
| **ADA (clinical cut-point: ≥35IU/l)** | 84.6%  (77.0-90.0) 99/117 | 84%  (65.4-93.6) 21/25 | 5.3  (3.2-8.7) | 0.183  (0.161-0.208) |

^*^p-values compare diagnostic tests for the indicated diagnostic accuracy measure e.g. sensitivity

^†^Denominators other than 124 definite/probable- and 27 non-TB patients indicate missing test results.

^‡^Youden’s index (the cut-point allowing the most correctly classified) and optimal ‘rule-out’ (the cut-point with the lowest LR-) cut-points for the uIFNγ (both the QFT-kit and Intergam-kits) were the same (QFT: 0.18 IU/l, Intergam: 44pg/ml) respectively

uIFNγ: unstimualted interferon gamma; ADA: adenosine deaminase; CI: Confidence interval; n/c: not calculatable

**ii) Analysis of diagnostic accuracy using only a single pericardial fluid liquid culture as the reference standard (pericardial fluid liquid culture positive for sensitivity and pericardial fluid culture negative for specificity calculations)**

Table S4. Diagnostic accuracy measures of Xpert MTB/RIF and the biomarkers uIFNγ and ADA using ROC-selected cut-points (pericardial fluid liquid culture positive for sensitivity and culture negative for specificity calculations). The reference standard was restricted to a single pericardial fluid liquid culture^†^.

|  | **Sensitivity**  **(95% CI)**  **(n/N)** | **Specificity**  **(95% CI)**  **(n/N)** | **Positive Likelihood ratio, LR+ (95% CI)** | **Negative Likelihood ratio, LR- (95% CI)** |
| --- | --- | --- | --- | --- |
| **Xpert MTB/RIF** | 59.6%  (45.3-72.4)  (28/47)^*^ | 69.0%  (58.6-77.7)  (60/87)^*^ | 1.9  (1.7-2.2) | 0.586 (0.521-0.660) |
| **uIFNγ (QFT-kit) (Cut-point: ≥0.18 IU/l)^‡^** | 97.9% (89.1-99.6) 47/48 ^*^p<0.001 | 32.6% (23.7-42.9) 29/89 ^*^p<0.001 | 1.45 (1.41-1.50) | 0.064  (0.008-0.522) |
| **uIFNγ (Intergam) (Cut-point: 44pg/ml)** | 93.8%  (83.2-97.9) 45/48 | 34.8%  (25.8-45.2) 31/89 | 1.44  (1.39-1.49) | 0.18  (0.083-0.39) |
| **ADA (clinical cut-point: ≥35IU/l)** | 95.7% (85.5-98.8) 44/46 | 39.6% (30.1-49.8) 36/91^**^ | 1.58 (1.52-1.64) | 0.110  (0.038-0.318) |

^†^A valid pericardial fluid culture result was only available for 134 patients (47 culture-positive and 87 culture-negative).

^*^p-values compare diagnostic tests for the indicated accuracy measure e.g. sensitivity.

P-values >0.05 are not shown

uIFNγ: unstimualted interferon gamma; ADA: adenosine deaminase; CI: Confidence interval; n/c: not calculatable

Table S5. Positive and negtive predictive value estimates for Xpert MTB/RIF, biomarkers uIFNγ and ADA, and clinical predictors using ROC selected cut-points, at differing prevalences of TB pericarditis (Low=10%, median=30% and high=50%) for a population of 1000.

| **Diagnostic**  **Test** | **Test sensitivity (%)** | **Test specificity (%)** | **TB prevalence amongst patients with pericardial effusions amendable to pericardiocentesis** | | | | | | |
| --- | --- | --- | --- | --- | --- | --- | --- | --- | --- |
|  |  |  | **10%** | | **30%** | | **50%** | | |
|  |  |  | **PPV**  **(%,95CI)** | **NPV (%,95CI)** | **PPV (%,95 I)** | **NPV (%,95 I)** | **PPV (%,95CI)** | **NPV (%,95CI)** |  |
| **Xpert MTB/RIF** | 63.8 | 100 | **100 (94.3-100)** | 96.1 (94.7-97.2) | 100 (98.0-100) | 86.6 (84.0-88.7) | **100 (98.8-100)** | 73.4 (70.0-76.6) |  |
| **uIFNγ ≥44 (Intergam)** | 95.7 | 96.3 | 74.2 (66.0-81.0) | 99.5 (98.8-99.8) | **93.3 (90.2-95.5)** | **97.7 (96.2-98.6)** | **96.9 (95.1-98.1)** | **94.8 (92.4-96.5)** |  |
| **ADA≥35IU/ml** | 95.7 | 84.0 | 39.9 (33.9-46.2) | 99.4 (98.6-99.8) | 71.9 (67.3-76.1) | **97.9 (96.4-98.7)** | 85.7 (82.5-88.3) | **94.1 (91.4-96.0)** |  |
| **ADA >107IU/ml** | 15.7 | 96 | 30.4 (19.6-43.9) | 91.1 (89.1-92.8) | 62.7 (51.4-72.8) | 72.7 (69.7-75.4) | 79.7 (70.7-86.5) | 53.2 (50.0-56.5) |  |
| **CPS >6.1** | 60.8 | 96.3 | 64.6 (54.6-73.5) | 95.7 (94.1-96.8) | 87.6 (82.4-91.4) | 85.1 (82.5-87.5) | **94.3 (91.2-96.3)** | 71.1 (67.5-74.4) |  |
| **CPS >3.5** | 91.9 | 81.5 | 35.6 (30.0-41.6) | 98.9 (97.9-99.4) | 68.0 (63.3-72.4) | 95.9 (94.0-97.2) | 83.2 (79.9-86.1) | 91.0 (87.9-93.3) |  |

PPV Positive predictive value; NPV Negative predictive value; TDIS Tygerberg TB Pericarditis Diagnostic Index;

PF ADA pericardial fluid adenosine deaminase; uIFN**γ** unstimulated interferon gamma, CPS: Clinical prediction score

**Supplementary Results - Figures**

Figure S1. Limit of detection of mycobacteria in pericardial fluid. H37Rv was spiked into 1-ml pericardial fluid aliquots. The proportion of positive samples for each CFU concentration out of 4 samples tested per concentration is shown.

**6. References**

1. White IR, Royston P, Wood AM. Multiple imputation using chained equations: Issues and guidance for practice. *Statistics in medicine*. 2011;30:377-399

2. Schisterman EF, Perkins NJ, Liu A, Bondell H. Optimal cut-point and its corresponding youden index to discriminate individuals using pooled blood samples. *Epidemiology*. 2005;16:73-81
